# Crystal structure of bis-*p*-anizidinegossypol with an unknown solvate

**DOI:** 10.1107/S2056989015020393

**Published:** 2015-11-04

**Authors:** Muhabbat T. Honkeldieva, Samat A. Talipov, Rishad Kunafiev, Bakhtiyar T. Ibragimov

**Affiliations:** a Institute of Bioorganich Chemistry, Mirzo Ulughbek Str., 83, Tashkent 100125, Uzbekistan

**Keywords:** crystal structure, gossypol, bis-*p*-anizidinegossypol, porous structure

## Abstract

The title compound, C_44_H_44_N_2_O_8_, (systematic name: 1,1′,6,6′-tetra­hydroxy-5,5′-diisopropyl-8,8′-bis­{[(4-meth­oxy­phen­yl)iminium­yl]meth­yl}-3,3′-dimethyl-2,2′-bi­naphthalene-7,7′-diolate) has been obtained by the addition of *p*-anizidine to gossypol dissolved in di­chloro­methane. In the solid state, the title compound exists in the enamine or quinoid form. The two naphthyl moieties are inclined to one another by 72.08 (5)°. The pendant phenyl rings are inclined at 22.26 (14) and 23.86 (13)° to the corresponding naphthyl rings. In the crystal, mol­ecules are incorporated into layers through inversion-related pairs of O—H⋯O inter­actions [graph sets *R*_2_^2^(20) and *R*_2_^2^(10)] and translation-related O—H⋯O inter­actions [graph set *C*(15)]. The packing of these layers in the crystal structure gives rise to channels in the [011] direction, with hydro­phobic inter­actions occurring between adjacent layers. The channels are 5–7 Å wide, and the void volume of each cell is 655 Å^3^, corresponding to 26.6% of the cell volume. Disordered guest mol­ecules, probably solvent and water mol­ecules, occupy these voids of the crystal; their contribution to the scattering was removed with the SQUEEZE routine [Spek (2015 ▸). *Acta Cryst*. C**71**, 9–18] of *PLATON* [Spek (2009 ▸). *Acta Cryst.* D**65**, 148–155].

## Abstract 2

In the solid-state, bis-*p*-anizidinegossypol exists in the enamine or quinoid form. The naphthyl moieties are close to perpendicular [dihedral angle = 72.08 (5)°]. In the crystal, mol­ecules are linked by O—H⋯O hydrogen bonds, generating layers which surround channels occupied by disordered guest mol­ecules.

## Chemical context   

Gossypol [2,2′-bis­(8-formyl-1,6,7-trihy­droxy-5-isopropyl-3-methyl­naphthalene)] is a yellow pigment of cotton seeds (Adams *et al.*, 1960 ▸). This compound was first isolated over 110 years ago (Marchlewski, 1899 ▸). Its study was initially important because the compound is associated with anti-nutritive or even toxic effects when cottonseed is overfed to animals. Many attempts have been made to either remove it from cottonseed or reduce its toxicity (Kenar, 2006 ▸). However, the compound also has a wide range of biological action, including anti-HIV (Jian Yang *et al.*, 2014 ▸), anti­cancer (Zhan *et al.*, 2009 ▸) and anti­fertility (Coutinho, 2002 ▸) effects. This inter­est has led to the synthesis and isolation of various gossypol derivatives, including many di­amine-based gossypol Schiff bases. Gossypol and its Schiff base formed with aniline have been previously reported to form inclusion compounds with many small organic compounds (Beketov *et al.*, 1994 ▸; Gdaniec *et al.*, 1996 ▸; Talipov *et al.*, 2004 ▸). Some gossypol polymorphs (referred to as the P3 polymorph; Ibragimov *et al.*, 1994 ▸), dianhydro­gossypol (Talipov *et al.*, 2009 ▸) and gossypol tetra­methyl ether (Honkeldieva *et al.*, 2015 ▸) form open-channel structures with channels of 5–8 Å width. In this report, we demonstrate that the Schiff base of gossypol with *p*-anizidine also forms an open-channel structure when the compound is crystallized from solutions in di­chloro­methane.
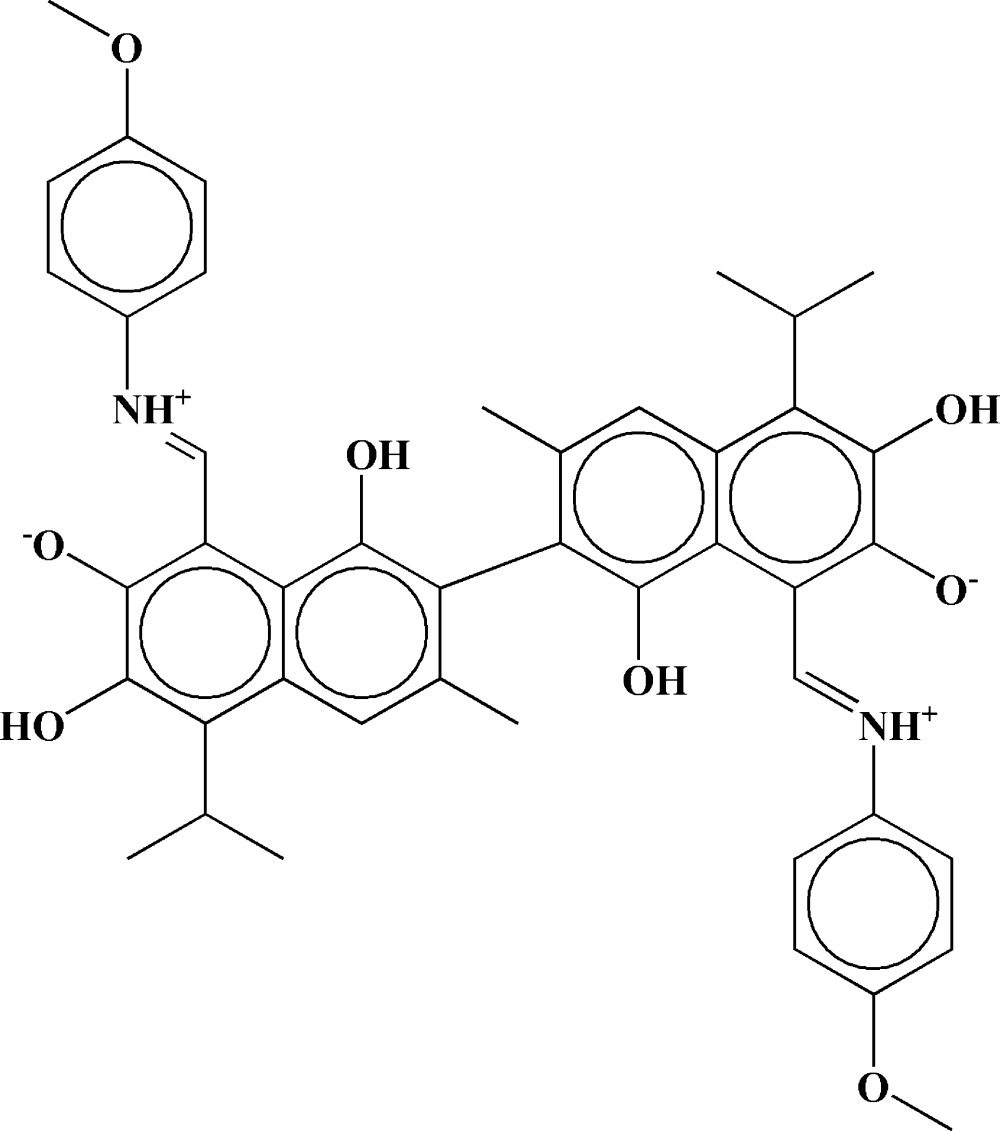


## Structural commentary   

**Figure 1:**
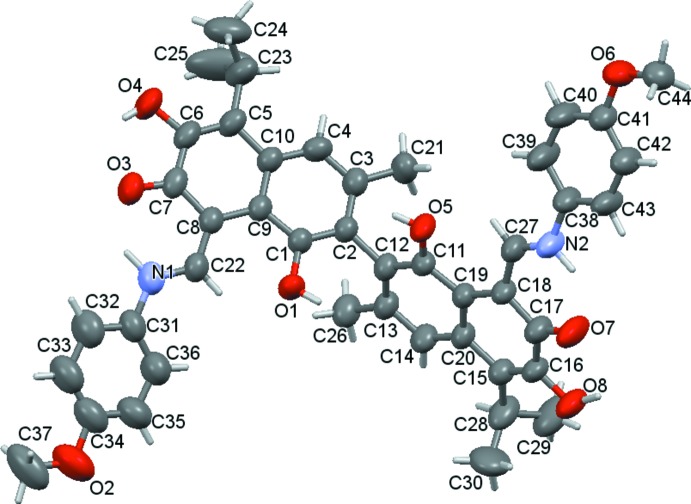
Mol­ecular structure of the title compound showing 50% probability displacement ellipsoids.

Gossypol can exist in one of the following tautomeric forms: aldehyde, quinoid and lactol (Adams *et al.*, 1960 ▸). In most solvents it is found in the aldehyde form; however, there are some reports that gossypol also exists in a pure lactol form (Reyes *et al.*, 1986 ▸) or as a dynamic equilibrium mixture of aldehyde and lactol forms in some highly polar solvents (Kamaev *et al.*, 1979 ▸). In the structure described here, the title compound is in the enamine or quinoid form. The highest symmetry which the title compound mol­ecule can possess is *C*_2_ (twofold axis perpendicular to the C2—C12 bond). However, bis-*p*-anizidinegossypol crystallizes in a triclinic (*P* 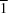) space group and the symmetry of the mol­ecule is not retained in the crystal. An *ORTEP* diagram of the mol­ecule and the atom numbering in the structure are given in Fig. 1 ▸.

The mol­ecule consists of four ring systems, two of which are naphthalene ring systems, and the other two are phenyl rings. The C1–C10 naphthyl unit is more planar then C11–C20 naphthyl one in which atoms C12, C16, C17, C18 and C19 deviate by 0.051 (3), 0.070 (3), 0.059 (3), 0.082 (3) and 0.054 (3) Å, respectively, from the mean plane. The two naphthyl moieties are inclined to one another by 72.08 (5)°. The phenyl rings are inclined at 22.26 (14) and 23.86 (13)° to the corresponding naphthyl rings. The bond lengths and angles are mostly in good agreement with those observed in the analogous fragments of the gossypol and dianilinegossypol mol­ecules (Gdaniec *et al.*, 1996 ▸; Talipov *et al.*, 2004 ▸). However, there are notable differences in the lengths of some bonds compared with typical gossypol values. Compared with the relatively short C6—C7 (C16—C17) aromatic ring bonds of gossypol mol­ecules (1.40 Å), the corresponding bonds in the bis-*p*-anizidinegossypol mol­ecule are longer with lengths of 1.446 (4) and 1.476 (4) Å. In addition, the N1—C22 [1.332 (4) Å] and N2—C27 [1.319 (4) Å] bonds are shorter than N1—C31 [1.433 (4) Å] and N2—C38 [1.441 (4) Å], respectively. Contrarily, C7=O3 [1.289 (3) Å] and C17=O7 [1.275 (3) Å] bonds are longer than typical standard values.

There are two intra­molecular hydrogen bonds in the mol­ecule. The N1—H1*A*⋯O3 (and N2—H2⋯O7) bond closes a six-membered ring C7—C8—C22—N1—H1*A*⋯O3 (and C17—C18—C27—N2—H3⋯O7), while the other type of hydrogen bond O4—H4⋯O3 (and O8—H8⋯O7) forms a five-membered ring C6—C7—O3⋯H4—O4 (and C16—C17—O7⋯H8—O8) (Table 1 ▸).

## Supra­molecular features   

**Figure 2:**
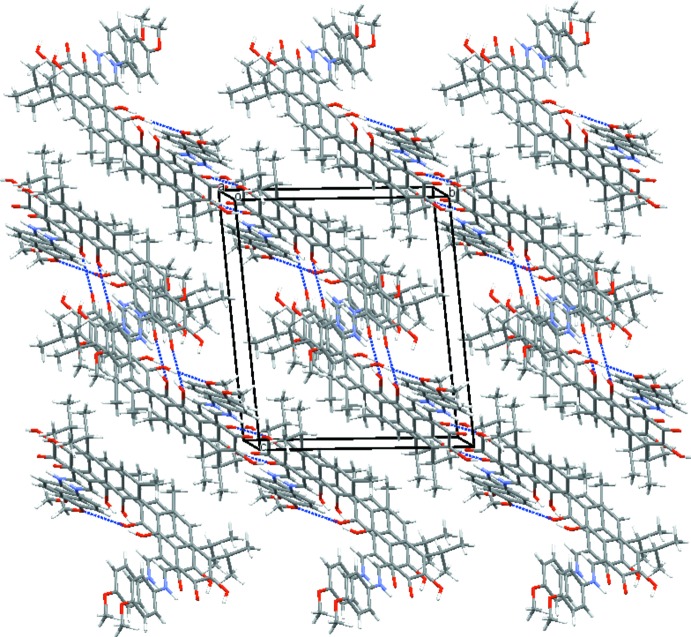
A portion of the crystal packing viewed approximately along the *a* axis.

The packing of the title mol­ecules in the crystal is shown in Fig. 2 ▸. Bis-*p*-anizidinegossypol mol­ecules are incorporated into centrosymmetric dimers typical for gossypol and dianilinogossypol crystal structures by means of a pair of inversion-related hydrogen bonds O5—H5⋯O3 [graph set 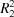
(20)]. By further centrosymmetric O8—H8⋯O7 hydrogen bonds [graph set 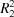
(10)], mol­ecules are associated into columns running in the [1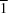 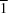] direction, as also seen for the dianilinegossypol clathrate with ethyl­acetate (Beketov *et al.*, 1994 ▸). A layer parallel to (01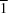) is formed by linking of the columns *via* translation-related hydrogen bonds O1—H1⋯O6 [graph set 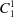
(15)] in the [100] direction. The layer features a O2⋯C7(−1 − *x*, 1 − *y*, 1 − *z*) contact [3.254 (4) Å] and a very weak aromatic π–π stacking inter­action with a *Cg*⋯*Cg*(−1 − *x*, 1 − *y*, 1 − *z*) distance of 4.182 (2) Å where *Cg* is the centroid of the C31–C36 ring. The packing of these layers in the crystal structure gives rise to wide ragged channels in the [011] direction. The stabilization of the crystal structure is supported by hydro­phobic inter­actions between adjacent layers. The channels in the structure are 5-7 Å wide and the void volume of each cell is 655 Å^3^, corresponding to 26.6% of the cell volume. Disordered solvated mol­ecules, probably solvent and water mol­ecules, occupy these voids of the crystal; their contribution to the scattering was removed with the SQUEEZE routine (Spek, 2015 ▸) of *PLATON* (Spek, 2009 ▸).

## Database survey   

A search in the Cambridge Structural Database (Version 5.36; Groom & Allen, 2014 ▸) indicated the presence of 198 entries for gossypol (137 entries) or gossypol derivatives. The 35 entries of revealed 50 entries for Schiff-base gossypol deriv­atives are related to dianilinegossypol clathrates and polymorphs. The dihedral angle between the naphthalene ring systems in the dianilinegossypol structures is in the range 78 to 90°. The dihedral angles between naphthalene ring systems and the corresponding benzyl rings of aniline substituents are in the range 4–49°. The dihedral angles between the naphthalene ring systems in the crystal structures of other Schiff base gossypol derivatives are in the range from *ca* 70 to 90°: IGAVAQ = 86.6°, LUHBIA = 89.6°, LUHBOG = 77.9°, MEXROY = 89.2°, MEXROY01 = 88.9°, NOQFIJ = 83.5°, POGHUF = 84.6°, QADQIX = 89.1°, SACXEB = 82.2°, TEFFEP = 70.6° and 83.4°, TIJNUX = 89.0°, XATPAK = 78.6°, VUXRIQ = 70.3°, VUXRIQ01 = 88.2° and 91.0° and YORNIW = 81.2°.

## Synthesis and crystallization   

Gossypol was obtained from the Experimental Plant of the Institute of Bioorganic Chemistry, Uzbekistan Academy of Sciences where it is produced from by-products of the cottonseed oil industry. To prepare the Schiff base complex, gossypol was mixed with *p*-anizidine in a 1:2 molar ratio in di­chloro­methane. This reaction solution was allowed to stand in the dark for some days, during which crystalline precipitates have been formed within the solution. The precipitate was recovered by filtration. Yield: 64%. After numerous attempts, a suitable crystal was selected from the precipitate and used for the diffraction study without additional recrystallization.

## Refinement   

Crystal data, data collection and structure refinement details are summarized in Table 2 ▸. The H atom of the hy­droxy substituent was located in an electron density map and its coordinates were freely refined with *U*_iso_ = 1.5*U*_eq_(O). C-bound H atoms were positioned geometrically and refined using a riding model, with *d*(C—H) = 0.93 Å and *U*_iso_ = 1.2*U*_eq_(C) for aromatic, *d*(C—H) = 0.98 Å and *U*iso = 1.2*U*_eq_ (C) for methine, *d*(C—H) = 0.96 Å and *U*_iso_ = 1.5*U*_eq_ (C) for methyl H atoms.

## Supplementary Material

Crystal structure: contains datablock(s) I. DOI: 10.1107/S2056989015020393/hb7516sup1.cif

Structure factors: contains datablock(s) I. DOI: 10.1107/S2056989015020393/hb7516Isup2.hkl

Supporting information file. DOI: 10.1107/S2056989015020393/hb7516Isup3.cml

CCDC reference: 1433643

Additional supporting information:  crystallographic information; 3D view; checkCIF report

## Figures and Tables

**Table 1:** Hydrogen-bond geometry (Å, °)

*D*—H⋯*A*	*D*—H	H⋯*A*	*D*⋯*A*	*D*—H⋯*A*
N1—H1*A*⋯O3	0.95 (5)	1.83 (5)	2.538 (4)	129 (4)
N2—H2⋯O7	0.92 (3)	1.87 (3)	2.550 (3)	129 (3)
O1—H1⋯O6^i^	0.91 (4)	2.13 (4)	2.912 (3)	144 (3)
O4—H4⋯O3	0.86 (3)	2.04 (4)	2.574 (4)	119 (3)
O5—H5⋯O3^ii^	0.82 (3)	1.98 (3)	2.684 (3)	143 (3)
O8—H8⋯O7	0.98 (3)	2.17 (3)	2.601 (3)	105 (2)
O8—H8⋯O7^iii^	0.98 (3)	1.83 (3)	2.757 (3)	158 (3)

Symmetry codes: (i) 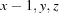; (ii) 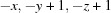; (iii) 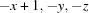.

**Table 2:** Experimental details

Crystal data	
Chemical formula	C_44_H_44_N_2_O_8_
*M*_r_	728.81
Crystal system, space group	Triclinic, *P* 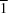
Temperature (K)	293
*a*, *b*, *c* (Å)	11.6622 (9), 14.0738 (11), 15.6906 (10)
α, β, γ (°)	82.472 (6), 84.831 (6), 75.009 (7)
*V* (Å^3^)	2462.0 (3)
*Z*	2
Radiation type	Cu *K*α
μ (mm^−1^)	0.55
Crystal size (mm)	0.40 × 0.32 × 0.27

Data collection	
Diffractometer	Oxford Diffraction Xcalibur Ruby
Absorption correction	Multi-scan (*CrysAlis PRO*; Oxford Diffraction, 2009 ▸)
*T*_min_, *T*_max_	0.811, 0.862
No. of measured, independent and observed [*I* > 2σ(*I*)] reflections	18952, 9019, 2706
*R*_int_	0.048
(sin θ/λ)_max_ (Å^−1^)	0.612

Refinement	
*R*[*F* ^2^ > 2σ(*F* ^2^)], *wR*(*F* ^2^), *S*	0.051, 0.124, 0.65
No. of reflections	9019
No. of parameters	519
H-atom treatment	H atoms treated by a mixture of independent and constrained refinement
Δρ_max_, Δρ_min_ (e Å^−3^)	0.13, −0.17

Computer programs: *CrysAlis PRO* (Oxford Diffraction, 2009 ▸), *SHELXS97* and *SHELXL97* (Sheldrick, 2008 ▸) and *OLEX2* (Dolomanov *et al.*, 2009 ▸).

## supplementary crystallographic information

### Crystal data

**Table:**

C_44_H_44_N_2_O_8_	*Z* = 2
*M**_r_* = 728.81	*F*(000) = 772
Triclinic, *P*1	*D*_x_ = 0.983 Mg m^−^^3^
*a* = 11.6622 (9) Å	Cu *K*α radiation, λ = 1.54184 Å
*b* = 14.0738 (11) Å	Cell parameters from 2564 reflections
*c* = 15.6906 (10) Å	θ = 3.9–70.6°
α = 82.472 (6)°	µ = 0.55 mm^−^^1^
β = 84.831 (6)°	*T* = 293 K
γ = 75.009 (7)°	Prism, broun
*V* = 2462.0 (3) Å^3^	0.40 × 0.32 × 0.27 mm

### Data collection

**Table:**

Oxford Diffraction Xcalibur Ruby diffractometer	9019 independent reflections
Radiation source: fine-focus sealed tube	2706 reflections with *I* > 2σ(*I*)
Graphite monochromator	*R*_int_ = 0.048
Detector resolution: 10.2576 pixels mm^-1^	θ_max_ = 70.6°, θ_min_ = 3.9°
ω scans	*h* = −14→13
Absorption correction: multi-scan (*CrysAlis PRO*; Oxford Diffraction, 2009)	*k* = −16→16
*T*_min_ = 0.811, *T*_max_ = 0.862	*l* = −18→16
18952 measured reflections	

### Refinement

**Table:**

Refinement on *F*^2^	Primary atom site location: structure-invariant direct methods
Least-squares matrix: full	Secondary atom site location: difference Fourier map
*R*[*F*^2^ > 2σ(*F*^2^)] = 0.051	Hydrogen site location: inferred from neighbouring sites
*wR*(*F*^2^) = 0.124	H atoms treated by a mixture of independent and constrained refinement
*S* = 0.65	*w* = 1/[σ^2^(*F*_o_^2^) + (0.0542*P*)^2^] where *P* = (*F*_o_^2^ + 2*F*_c_^2^)/3
9019 reflections	(Δ/σ)_max_ < 0.001
519 parameters	Δρ_max_ = 0.13 e Å^−^^3^
0 restraints	Δρ_min_ = −0.17 e Å^−^^3^

### Special details

**Table:**

Experimental. Absorption correction: CrysAlisPro, Oxford Diffraction (2009), Empirical absorption correction using spherical harmonics, implemented in SCALE3 ABSPACK scaling algorithm.
Geometry. All e.s.d.'s (except the e.s.d. in the dihedral angle between two l.s. planes) are estimated using the full covariance matrix. The cell e.s.d.'s are taken into account individually in the estimation of e.s.d.'s in distances, angles and torsion angles; correlations between e.s.d.'s in cell parameters are only used when they are defined by crystal symmetry. An approximate (isotropic) treatment of cell e.s.d.'s is used for estimating e.s.d.'s involving l.s. planes.
Refinement. Refinement of *F*^2^ against ALL reflections. The weighted *R*-factor *wR* and goodness of fit *S* are based on *F*^2^, conventional *R*-factors *R* are based on *F*, with *F* set to zero for negative *F*^2^. The threshold expression of *F*^2^ > σ(*F*^2^) is used only for calculating *R*-factors(gt) *etc*. and is not relevant to the choice of reflections for refinement. *R*-factors based on *F*^2^ are statistically about twice as large as those based on *F*, and *R*- factors based on ALL data will be even larger.

### Fractional atomic coordinates and isotropic or equivalent isotropic displacement parameters (Å^2^)

**Table:**

	*x*	*y*	*z*	*U*_iso_*/*U*_eq_	
O1	−0.06997 (18)	0.40724 (17)	0.33397 (13)	0.0668 (6)	
O2	−0.6101 (2)	0.2359 (2)	0.58343 (19)	0.1250 (10)	
O3	−0.23580 (18)	0.62089 (16)	0.58330 (12)	0.0774 (6)	
O4	−0.1425 (2)	0.7699 (2)	0.55783 (17)	0.0923 (8)	
O5	0.27695 (17)	0.35008 (18)	0.24986 (14)	0.0741 (7)	
O6	0.97592 (18)	0.21416 (17)	0.27160 (14)	0.0886 (7)	
O7	0.46897 (19)	0.06734 (18)	0.07019 (15)	0.1002 (9)	
O8	0.3149 (2)	0.06336 (18)	−0.03842 (14)	0.0887 (8)	
N1	−0.2902 (2)	0.4750 (2)	0.53257 (17)	0.0736 (8)	
N2	0.5550 (2)	0.1627 (2)	0.16491 (18)	0.0824 (9)	
C1	−0.0203 (2)	0.4872 (2)	0.32283 (17)	0.0543 (7)	
C2	0.0593 (2)	0.4959 (2)	0.25382 (17)	0.0546 (8)	
C3	0.1135 (3)	0.5752 (2)	0.24359 (18)	0.0657 (9)	
C4	0.0827 (3)	0.6422 (2)	0.30335 (19)	0.0677 (9)	
H4A	0.1188	0.6946	0.2968	0.081*	
C5	−0.0267 (3)	0.7112 (2)	0.43314 (19)	0.0680 (9)	
C6	−0.1066 (3)	0.7018 (2)	0.4998 (2)	0.0681 (9)	
C7	−0.1641 (3)	0.6211 (2)	0.51583 (18)	0.0627 (9)	
C8	−0.1427 (2)	0.5498 (2)	0.45576 (17)	0.0570 (8)	
C9	−0.0561 (2)	0.5568 (2)	0.38381 (17)	0.0537 (8)	
C10	0.0002 (2)	0.6367 (2)	0.37361 (18)	0.0600 (8)	
C11	0.1982 (2)	0.3535 (2)	0.18905 (16)	0.0563 (8)	
C12	0.0890 (2)	0.4206 (2)	0.19037 (17)	0.0587 (8)	
C13	0.0074 (2)	0.4204 (2)	0.12890 (17)	0.0615 (8)	
C14	0.0405 (3)	0.3496 (2)	0.07254 (17)	0.0688 (9)	
H14	−0.0149	0.3470	0.0344	0.083*	
C15	0.1802 (2)	0.2079 (2)	0.00811 (18)	0.0623 (8)	
C16	0.2827 (3)	0.1384 (2)	0.01332 (18)	0.0677 (9)	
C17	0.3742 (3)	0.1370 (3)	0.07291 (19)	0.0730 (10)	
C18	0.3505 (2)	0.2143 (2)	0.12572 (17)	0.0601 (8)	
C19	0.2357 (2)	0.2845 (2)	0.12808 (17)	0.0571 (8)	
C20	0.1511 (2)	0.2814 (2)	0.06846 (17)	0.0591 (8)	
C21	0.1981 (3)	0.5900 (2)	0.16700 (19)	0.0862 (11)	
H21A	0.2164	0.6529	0.1662	0.129*	
H21B	0.1616	0.5886	0.1149	0.129*	
H21C	0.2700	0.5381	0.1713	0.129*	
C22	−0.2114 (3)	0.4812 (2)	0.46653 (19)	0.0642 (8)	
H22	−0.2017	0.4374	0.4254	0.077*	
C23	0.0276 (4)	0.7993 (3)	0.4206 (2)	0.0993 (13)	
H23	0.0914	0.7849	0.3755	0.119*	
C24	0.0868 (4)	0.8144 (3)	0.4976 (3)	0.1269 (15)	
H24A	0.1543	0.8405	0.4785	0.190*	
H24B	0.1126	0.7522	0.5323	0.190*	
H24C	0.0313	0.8601	0.5312	0.190*	
C25	−0.0552 (5)	0.8930 (4)	0.3869 (3)	0.192 (3)	
H25A	−0.0664	0.8912	0.3274	0.288*	
H25B	−0.0226	0.9478	0.3925	0.288*	
H25C	−0.1304	0.9008	0.4192	0.288*	
C26	−0.1129 (3)	0.4922 (2)	0.12655 (19)	0.0819 (10)	
H26A	−0.1553	0.4803	0.0810	0.123*	
H26B	−0.1035	0.5586	0.1166	0.123*	
H26C	−0.1567	0.4836	0.1806	0.123*	
C27	0.4492 (3)	0.2255 (2)	0.16703 (18)	0.0742 (10)	
H27	0.4385	0.2801	0.1972	0.089*	
C28	0.0952 (3)	0.2073 (3)	−0.0607 (2)	0.0935 (13)	
H28	0.0313	0.2681	−0.0592	0.112*	
C29	0.1528 (3)	0.2089 (3)	−0.1511 (2)	0.1217 (16)	
H29A	0.1918	0.2619	−0.1618	0.183*	
H29B	0.0930	0.2187	−0.1919	0.183*	
H29C	0.2101	0.1471	−0.1571	0.183*	
C30	0.0377 (4)	0.1211 (4)	−0.0378 (3)	0.1432 (19)	
H30A	−0.0126	0.1310	0.0138	0.215*	
H30B	0.0985	0.0606	−0.0287	0.215*	
H30C	−0.0089	0.1174	−0.0841	0.215*	
C31	−0.3701 (3)	0.4116 (3)	0.5449 (2)	0.0736 (9)	
C32	−0.4309 (3)	0.4051 (3)	0.6236 (2)	0.1032 (13)	
H32	−0.4178	0.4399	0.6668	0.124*	
C33	−0.5130 (3)	0.3461 (3)	0.6395 (3)	0.1062 (13)	
H33	−0.5537	0.3408	0.6930	0.127*	
C34	−0.5315 (3)	0.2971 (3)	0.5753 (3)	0.0971 (12)	
C35	−0.4745 (3)	0.3059 (3)	0.4958 (3)	0.1026 (12)	
H35	−0.4903	0.2730	0.4523	0.123*	
C36	−0.3941 (3)	0.3627 (3)	0.4797 (2)	0.0961 (12)	
H36	−0.3558	0.3686	0.4254	0.115*	
C37	−0.6735 (3)	0.2272 (4)	0.6640 (3)	0.1379 (18)	
H37A	−0.7292	0.1883	0.6609	0.207*	
H37B	−0.7155	0.2919	0.6782	0.207*	
H37C	−0.6188	0.1956	0.7074	0.207*	
C38	0.6624 (3)	0.1761 (3)	0.1948 (2)	0.0750 (10)	
C39	0.6640 (3)	0.2563 (3)	0.2352 (2)	0.1141 (15)	
H39	0.5938	0.3032	0.2470	0.137*	
C40	0.7716 (3)	0.2665 (3)	0.2581 (2)	0.1103 (14)	
H40	0.7740	0.3230	0.2821	0.132*	
C41	0.8741 (3)	0.1953 (3)	0.24610 (19)	0.0739 (10)	
C42	0.8717 (3)	0.1150 (3)	0.2073 (2)	0.0886 (11)	
H42	0.9415	0.0669	0.1976	0.106*	
C43	0.7637 (3)	0.1053 (3)	0.1821 (2)	0.0935 (12)	
H43	0.7616	0.0499	0.1565	0.112*	
C44	1.0825 (3)	0.1378 (2)	0.2661 (2)	0.0842 (10)	
H44A	1.1459	0.1576	0.2882	0.126*	
H44B	1.1024	0.1261	0.2070	0.126*	
H44C	1.0713	0.0783	0.2993	0.126*	
H1A	−0.302 (4)	0.518 (4)	0.577 (3)	0.20 (2)*	
H2	0.571 (3)	0.109 (2)	0.1344 (19)	0.095 (11)*	
H1	−0.023 (3)	0.352 (3)	0.313 (2)	0.133 (16)*	
H4	−0.184 (3)	0.745 (3)	0.598 (2)	0.122 (18)*	
H5	0.243 (2)	0.381 (2)	0.2898 (17)	0.071 (10)*	
H8	0.397 (3)	0.023 (3)	−0.037 (2)	0.124 (14)*	

### Atomic displacement parameters (Å^2^)

**Table:**

	*U*^11^	*U*^22^	*U*^33^	*U*^12^	*U*^13^	*U*^23^
O1	0.0682 (13)	0.0589 (16)	0.0781 (14)	−0.0206 (12)	0.0069 (11)	−0.0238 (12)
O2	0.099 (2)	0.146 (3)	0.140 (3)	−0.063 (2)	−0.0104 (18)	0.017 (2)
O3	0.0750 (14)	0.0936 (19)	0.0625 (13)	−0.0130 (12)	0.0044 (11)	−0.0264 (12)
O4	0.0997 (19)	0.099 (2)	0.0850 (18)	−0.0209 (16)	0.0106 (15)	−0.0536 (16)
O5	0.0574 (13)	0.0986 (19)	0.0710 (14)	−0.0096 (12)	−0.0063 (11)	−0.0454 (14)
O6	0.0646 (14)	0.0966 (19)	0.1167 (18)	−0.0211 (14)	−0.0188 (13)	−0.0442 (15)
O7	0.0664 (14)	0.106 (2)	0.130 (2)	0.0146 (14)	−0.0295 (13)	−0.0754 (16)
O8	0.0720 (15)	0.0960 (19)	0.1022 (17)	0.0015 (14)	−0.0224 (13)	−0.0600 (15)
N1	0.0690 (18)	0.085 (2)	0.0626 (18)	−0.0132 (16)	0.0030 (15)	−0.0088 (16)
N2	0.0558 (17)	0.091 (2)	0.106 (2)	−0.0032 (16)	−0.0107 (14)	−0.0588 (19)
C1	0.0517 (17)	0.050 (2)	0.0596 (19)	−0.0086 (15)	−0.0007 (15)	−0.0118 (15)
C2	0.0550 (17)	0.055 (2)	0.0539 (18)	−0.0107 (16)	0.0007 (15)	−0.0152 (15)
C3	0.0634 (19)	0.074 (2)	0.0596 (19)	−0.0141 (18)	0.0024 (15)	−0.0169 (17)
C4	0.072 (2)	0.056 (2)	0.080 (2)	−0.0191 (17)	−0.0099 (18)	−0.0172 (18)
C5	0.083 (2)	0.063 (2)	0.061 (2)	−0.0160 (18)	−0.0017 (17)	−0.0223 (17)
C6	0.067 (2)	0.070 (2)	0.067 (2)	−0.0036 (18)	−0.0080 (17)	−0.0309 (18)
C7	0.0558 (19)	0.068 (2)	0.056 (2)	0.0051 (17)	−0.0085 (16)	−0.0172 (17)
C8	0.0521 (17)	0.060 (2)	0.0576 (18)	−0.0088 (16)	−0.0031 (15)	−0.0136 (16)
C9	0.0474 (16)	0.054 (2)	0.0570 (18)	−0.0038 (15)	−0.0039 (14)	−0.0151 (15)
C10	0.0609 (19)	0.057 (2)	0.063 (2)	−0.0113 (16)	−0.0017 (16)	−0.0186 (16)
C11	0.0543 (18)	0.067 (2)	0.0513 (17)	−0.0173 (16)	0.0010 (14)	−0.0202 (15)
C12	0.0559 (19)	0.065 (2)	0.0545 (18)	−0.0082 (16)	0.0002 (15)	−0.0191 (16)
C13	0.0612 (19)	0.061 (2)	0.0595 (18)	−0.0050 (16)	−0.0022 (15)	−0.0184 (16)
C14	0.064 (2)	0.085 (3)	0.0593 (19)	−0.0121 (18)	−0.0081 (15)	−0.0252 (18)
C15	0.0551 (18)	0.066 (2)	0.067 (2)	−0.0084 (17)	−0.0084 (15)	−0.0228 (17)
C16	0.062 (2)	0.074 (2)	0.074 (2)	−0.0143 (18)	−0.0047 (16)	−0.0385 (18)
C17	0.061 (2)	0.080 (3)	0.082 (2)	−0.0069 (19)	−0.0089 (17)	−0.040 (2)
C18	0.0492 (17)	0.070 (2)	0.0641 (19)	−0.0088 (16)	−0.0069 (14)	−0.0295 (17)
C19	0.0486 (17)	0.066 (2)	0.0601 (18)	−0.0123 (15)	0.0012 (14)	−0.0242 (16)
C20	0.0532 (18)	0.064 (2)	0.0594 (18)	−0.0062 (16)	−0.0010 (14)	−0.0234 (16)
C21	0.086 (2)	0.088 (3)	0.090 (2)	−0.034 (2)	0.026 (2)	−0.028 (2)
C22	0.0582 (18)	0.066 (2)	0.066 (2)	−0.0066 (17)	0.0035 (16)	−0.0186 (16)
C23	0.133 (3)	0.092 (3)	0.091 (3)	−0.053 (3)	0.019 (2)	−0.043 (2)
C24	0.125 (3)	0.111 (4)	0.168 (4)	−0.061 (3)	−0.048 (3)	−0.013 (3)
C25	0.273 (7)	0.104 (4)	0.234 (6)	−0.104 (5)	−0.152 (5)	0.065 (4)
C26	0.068 (2)	0.094 (3)	0.079 (2)	−0.0016 (19)	−0.0089 (17)	−0.026 (2)
C27	0.058 (2)	0.083 (3)	0.085 (2)	−0.0089 (18)	−0.0003 (17)	−0.0433 (19)
C28	0.076 (2)	0.118 (3)	0.086 (3)	0.005 (2)	−0.026 (2)	−0.052 (2)
C29	0.148 (4)	0.126 (4)	0.082 (3)	0.007 (3)	−0.035 (3)	−0.042 (2)
C30	0.101 (3)	0.199 (5)	0.166 (4)	−0.074 (3)	−0.020 (3)	−0.070 (4)
C31	0.060 (2)	0.085 (3)	0.073 (2)	−0.0167 (19)	0.0062 (18)	−0.0060 (19)
C32	0.099 (3)	0.129 (4)	0.080 (3)	−0.035 (3)	0.005 (2)	−0.002 (2)
C33	0.094 (3)	0.134 (4)	0.093 (3)	−0.046 (3)	0.010 (2)	0.006 (3)
C34	0.074 (2)	0.105 (4)	0.109 (3)	−0.032 (2)	0.002 (2)	0.012 (3)
C35	0.085 (3)	0.112 (4)	0.121 (3)	−0.045 (3)	0.003 (2)	−0.015 (3)
C36	0.086 (3)	0.114 (3)	0.094 (3)	−0.036 (2)	0.014 (2)	−0.025 (2)
C37	0.094 (3)	0.185 (5)	0.128 (4)	−0.057 (3)	−0.009 (3)	0.054 (3)
C38	0.0506 (18)	0.088 (3)	0.093 (2)	−0.0087 (18)	−0.0132 (16)	−0.047 (2)
C39	0.069 (2)	0.121 (3)	0.164 (4)	−0.003 (2)	−0.015 (2)	−0.097 (3)
C40	0.071 (2)	0.118 (3)	0.161 (4)	−0.016 (2)	−0.021 (2)	−0.088 (3)
C41	0.061 (2)	0.084 (3)	0.084 (2)	−0.0150 (19)	−0.0073 (17)	−0.037 (2)
C42	0.056 (2)	0.090 (3)	0.125 (3)	−0.0051 (19)	−0.0157 (19)	−0.051 (2)
C43	0.063 (2)	0.087 (3)	0.140 (3)	−0.007 (2)	−0.015 (2)	−0.068 (2)
C44	0.064 (2)	0.089 (3)	0.105 (3)	−0.020 (2)	−0.0120 (19)	−0.023 (2)

### Geometric parameters (Å, º)

**Table:**

O1—C1	1.379 (3)	C21—H21B	0.9600
O1—H1	0.91 (4)	C21—H21C	0.9600
O2—C34	1.398 (4)	C22—H22	0.9300
O2—C37	1.413 (4)	C23—H23	0.9800
O3—C7	1.289 (3)	C23—C24	1.508 (4)
O4—C6	1.371 (3)	C23—C25	1.484 (5)
O4—H4	0.86 (3)	C24—H24A	0.9600
O5—C11	1.371 (3)	C24—H24B	0.9600
O5—H5	0.82 (3)	C24—H24C	0.9600
O6—C41	1.384 (3)	C25—H25A	0.9600
O6—C44	1.422 (3)	C25—H25B	0.9600
O7—C17	1.275 (3)	C25—H25C	0.9600
O8—C16	1.371 (3)	C26—H26A	0.9600
O8—H8	0.98 (3)	C26—H26B	0.9600
N1—C22	1.332 (4)	C26—H26C	0.9600
N1—C31	1.433 (4)	C27—H27	0.9300
N1—H1A	0.95 (5)	C28—H28	0.9800
N2—C27	1.319 (4)	C28—C29	1.514 (4)
N2—C38	1.441 (4)	C28—C30	1.521 (5)
N2—H2	0.92 (3)	C29—H29A	0.9600
C1—C2	1.376 (3)	C29—H29B	0.9600
C1—C9	1.417 (3)	C29—H29C	0.9600
C2—C3	1.403 (4)	C30—H30A	0.9600
C2—C12	1.502 (4)	C30—H30B	0.9600
C3—C4	1.376 (4)	C30—H30C	0.9600
C3—C21	1.513 (4)	C31—C32	1.373 (4)
C4—H4A	0.9300	C31—C36	1.391 (4)
C4—C10	1.404 (4)	C32—H32	0.9300
C5—C6	1.353 (4)	C32—C33	1.409 (5)
C5—C10	1.450 (4)	C33—H33	0.9300
C5—C23	1.516 (5)	C33—C34	1.354 (5)
C6—C7	1.446 (4)	C34—C35	1.367 (5)
C7—C8	1.426 (4)	C35—H35	0.9300
C8—C9	1.456 (4)	C35—C36	1.368 (5)
C8—C22	1.390 (4)	C36—H36	0.9300
C9—C10	1.428 (4)	C37—H37A	0.9600
C11—C12	1.376 (4)	C37—H37B	0.9600
C11—C19	1.414 (3)	C37—H37C	0.9600
C12—C13	1.415 (4)	C38—C39	1.369 (4)
C13—C14	1.376 (3)	C38—C43	1.353 (4)
C13—C26	1.502 (4)	C39—H39	0.9300
C14—H14	0.9300	C39—C40	1.383 (4)
C14—C20	1.397 (4)	C40—H40	0.9300
C15—C16	1.336 (4)	C40—C41	1.364 (4)
C15—C20	1.449 (3)	C41—C42	1.359 (4)
C15—C28	1.532 (4)	C42—H42	0.9300
C16—C17	1.476 (4)	C42—C43	1.398 (4)
C17—C18	1.409 (4)	C43—H43	0.9300
C18—C19	1.445 (4)	C44—H44A	0.9600
C18—C27	1.423 (4)	C44—H44B	0.9600
C19—C20	1.431 (4)	C44—H44C	0.9600
C21—H21A	0.9600		
			
C1—O1—H1	114 (2)	C23—C24—H24A	109.5
C34—O2—C37	116.4 (4)	C23—C24—H24B	109.5
C6—O4—H4	107 (3)	C23—C24—H24C	109.5
C11—O5—H5	110 (2)	H24A—C24—H24B	109.5
C41—O6—C44	116.8 (2)	H24A—C24—H24C	109.5
C16—O8—H8	117 (2)	H24B—C24—H24C	109.5
C22—N1—C31	126.7 (3)	C23—C25—H25A	109.5
C22—N1—H1A	121 (3)	C23—C25—H25B	109.5
C31—N1—H1A	112 (3)	C23—C25—H25C	109.5
C27—N2—C38	126.5 (3)	H25A—C25—H25B	109.5
C27—N2—H2	122 (2)	H25A—C25—H25C	109.5
C38—N2—H2	111 (2)	H25B—C25—H25C	109.5
O1—C1—C9	117.2 (3)	C13—C26—H26A	109.5
C2—C1—O1	119.0 (2)	C13—C26—H26B	109.5
C2—C1—C9	123.8 (3)	C13—C26—H26C	109.5
C1—C2—C3	119.4 (3)	H26A—C26—H26B	109.5
C1—C2—C12	120.3 (3)	H26A—C26—H26C	109.5
C3—C2—C12	120.2 (3)	H26B—C26—H26C	109.5
C2—C3—C21	121.4 (3)	N2—C27—C18	123.6 (3)
C4—C3—C2	117.8 (3)	N2—C27—H27	118.2
C4—C3—C21	120.7 (3)	C18—C27—H27	118.2
C3—C4—H4A	117.8	C15—C28—H28	106.9
C3—C4—C10	124.3 (3)	C29—C28—C15	113.3 (3)
C10—C4—H4A	117.8	C29—C28—H28	106.9
C6—C5—C10	117.5 (3)	C29—C28—C30	112.2 (3)
C6—C5—C23	120.2 (3)	C30—C28—C15	110.2 (3)
C10—C5—C23	122.3 (3)	C30—C28—H28	106.9
O4—C6—C7	113.5 (3)	C28—C29—H29A	109.5
C5—C6—O4	122.6 (3)	C28—C29—H29B	109.5
C5—C6—C7	123.9 (3)	C28—C29—H29C	109.5
O3—C7—C6	116.7 (3)	H29A—C29—H29B	109.5
O3—C7—C8	124.1 (3)	H29A—C29—H29C	109.5
C8—C7—C6	119.2 (3)	H29B—C29—H29C	109.5
C7—C8—C9	118.4 (3)	C28—C30—H30A	109.5
C22—C8—C7	117.8 (3)	C28—C30—H30B	109.5
C22—C8—C9	123.8 (3)	C28—C30—H30C	109.5
C1—C9—C8	124.3 (3)	H30A—C30—H30B	109.5
C1—C9—C10	116.4 (3)	H30A—C30—H30C	109.5
C10—C9—C8	119.2 (2)	H30B—C30—H30C	109.5
C4—C10—C5	120.2 (3)	C32—C31—N1	117.3 (3)
C4—C10—C9	118.1 (3)	C32—C31—C36	119.3 (4)
C9—C10—C5	121.7 (3)	C36—C31—N1	123.2 (3)
O5—C11—C12	120.1 (2)	C31—C32—H32	119.8
O5—C11—C19	116.3 (2)	C31—C32—C33	120.4 (4)
C12—C11—C19	123.6 (2)	C33—C32—H32	119.8
C11—C12—C2	120.2 (2)	C32—C33—H33	120.6
C11—C12—C13	119.1 (2)	C34—C33—C32	118.7 (4)
C13—C12—C2	120.7 (2)	C34—C33—H33	120.6
C12—C13—C26	121.7 (2)	C33—C34—O2	124.0 (4)
C14—C13—C12	117.6 (3)	C33—C34—C35	121.3 (4)
C14—C13—C26	120.7 (3)	C35—C34—O2	114.7 (4)
C13—C14—H14	117.6	C34—C35—H35	119.7
C13—C14—C20	124.8 (3)	C34—C35—C36	120.5 (4)
C20—C14—H14	117.6	C36—C35—H35	119.7
C16—C15—C20	119.2 (3)	C31—C36—H36	120.1
C16—C15—C28	119.3 (3)	C35—C36—C31	119.7 (4)
C20—C15—C28	121.4 (3)	C35—C36—H36	120.1
O8—C16—C17	113.5 (3)	O2—C37—H37A	109.5
C15—C16—O8	123.2 (3)	O2—C37—H37B	109.5
C15—C16—C17	123.4 (3)	O2—C37—H37C	109.5
O7—C17—C16	116.9 (3)	H37A—C37—H37B	109.5
O7—C17—C18	126.0 (3)	H37A—C37—H37C	109.5
C18—C17—C16	117.1 (3)	H37B—C37—H37C	109.5
C17—C18—C19	120.7 (2)	C39—C38—N2	122.5 (3)
C17—C18—C27	116.1 (2)	C43—C38—N2	117.3 (3)
C27—C18—C19	122.7 (2)	C43—C38—C39	120.2 (3)
C11—C19—C18	124.1 (2)	C38—C39—H39	120.5
C11—C19—C20	117.1 (2)	C38—C39—C40	119.0 (3)
C20—C19—C18	118.7 (2)	C40—C39—H39	120.5
C14—C20—C15	122.2 (3)	C39—C40—H40	119.5
C14—C20—C19	117.6 (2)	C41—C40—C39	121.1 (3)
C19—C20—C15	120.2 (2)	C41—C40—H40	119.5
C3—C21—H21A	109.5	C40—C41—O6	115.6 (3)
C3—C21—H21B	109.5	C42—C41—O6	124.7 (3)
C3—C21—H21C	109.5	C42—C41—C40	119.5 (3)
H21A—C21—H21B	109.5	C41—C42—H42	120.2
H21A—C21—H21C	109.5	C41—C42—C43	119.6 (3)
H21B—C21—H21C	109.5	C43—C42—H42	120.2
N1—C22—C8	123.5 (3)	C38—C43—C42	120.3 (3)
N1—C22—H22	118.3	C38—C43—H43	119.8
C8—C22—H22	118.3	C42—C43—H43	119.8
C5—C23—H23	105.6	O6—C44—H44A	109.5
C24—C23—C5	115.0 (3)	O6—C44—H44B	109.5
C24—C23—H23	105.6	O6—C44—H44C	109.5
C25—C23—C5	113.6 (4)	H44A—C44—H44B	109.5
C25—C23—H23	105.6	H44A—C44—H44C	109.5
C25—C23—C24	110.6 (3)	H44B—C44—H44C	109.5
			
O1—C1—C2—C3	−177.8 (3)	C12—C11—C19—C18	179.5 (3)
O1—C1—C2—C12	1.6 (4)	C12—C11—C19—C20	−4.0 (5)
O1—C1—C9—C8	−1.1 (4)	C12—C13—C14—C20	−3.7 (5)
O1—C1—C9—C10	177.2 (2)	C13—C14—C20—C15	179.4 (3)
O2—C34—C35—C36	−179.2 (4)	C13—C14—C20—C19	1.0 (5)
O3—C7—C8—C9	−177.9 (2)	C15—C16—C17—O7	−178.8 (3)
O3—C7—C8—C22	6.1 (4)	C15—C16—C17—C18	−0.6 (5)
O4—C6—C7—O3	−3.1 (4)	C16—C15—C20—C14	−173.1 (3)
O4—C6—C7—C8	174.5 (3)	C16—C15—C20—C19	5.2 (5)
O5—C11—C12—C2	5.0 (4)	C16—C15—C28—C29	−55.0 (5)
O5—C11—C12—C13	−177.7 (3)	C16—C15—C28—C30	71.5 (4)
O5—C11—C19—C18	−1.5 (5)	C16—C17—C18—C19	7.1 (5)
O5—C11—C19—C20	175.0 (3)	C16—C17—C18—C27	−165.5 (3)
O6—C41—C42—C43	−178.1 (3)	C17—C18—C19—C11	169.1 (3)
O7—C17—C18—C19	−174.9 (3)	C17—C18—C19—C20	−7.3 (5)
O7—C17—C18—C27	12.5 (5)	C17—C18—C27—N2	−6.9 (5)
O8—C16—C17—O7	−1.0 (5)	C18—C19—C20—C14	179.5 (3)
O8—C16—C17—C18	177.2 (3)	C18—C19—C20—C15	1.1 (5)
N1—C31—C32—C33	178.5 (3)	C19—C11—C12—C2	−176.0 (3)
N1—C31—C36—C35	−177.9 (3)	C19—C11—C12—C13	1.3 (5)
N2—C38—C39—C40	−176.7 (4)	C19—C18—C27—N2	−179.3 (3)
N2—C38—C43—C42	178.2 (3)	C20—C15—C16—O8	176.9 (3)
C1—C2—C3—C4	−0.9 (4)	C20—C15—C16—C17	−5.5 (5)
C1—C2—C3—C21	−177.7 (3)	C20—C15—C28—C29	125.8 (3)
C1—C2—C12—C11	−107.7 (3)	C20—C15—C28—C30	−107.6 (4)
C1—C2—C12—C13	75.0 (4)	C21—C3—C4—C10	176.5 (3)
C1—C9—C10—C4	2.1 (4)	C22—N1—C31—C32	169.9 (3)
C1—C9—C10—C5	−178.4 (3)	C22—N1—C31—C36	−14.6 (5)
C2—C1—C9—C8	178.3 (3)	C22—C8—C9—C1	−8.7 (4)
C2—C1—C9—C10	−3.4 (4)	C22—C8—C9—C10	173.1 (3)
C2—C3—C4—C10	−0.3 (5)	C23—C5—C6—O4	0.4 (5)
C2—C12—C13—C14	179.8 (3)	C23—C5—C6—C7	179.0 (3)
C2—C12—C13—C26	−2.3 (5)	C23—C5—C10—C4	2.7 (5)
C3—C2—C12—C11	71.7 (4)	C23—C5—C10—C9	−176.8 (3)
C3—C2—C12—C13	−105.6 (3)	C26—C13—C14—C20	178.4 (3)
C3—C4—C10—C5	−179.9 (3)	C27—N2—C38—C39	4.3 (6)
C3—C4—C10—C9	−0.3 (4)	C27—N2—C38—C43	−176.1 (3)
C5—C6—C7—O3	178.2 (3)	C27—C18—C19—C11	−18.8 (5)
C5—C6—C7—C8	−4.3 (5)	C27—C18—C19—C20	164.8 (3)
C6—C5—C10—C4	−179.8 (3)	C28—C15—C16—O8	−2.3 (5)
C6—C5—C10—C9	0.7 (4)	C28—C15—C16—C17	175.3 (3)
C6—C5—C23—C24	54.8 (5)	C28—C15—C20—C14	6.0 (5)
C6—C5—C23—C25	−74.1 (4)	C28—C15—C20—C19	−175.6 (3)
C6—C7—C8—C9	4.7 (4)	C31—N1—C22—C8	175.1 (3)
C6—C7—C8—C22	−171.3 (3)	C31—C32—C33—C34	−0.8 (6)
C7—C8—C9—C1	175.5 (3)	C32—C31—C36—C35	−2.5 (6)
C7—C8—C9—C10	−2.7 (4)	C32—C33—C34—O2	179.6 (4)
C7—C8—C22—N1	−4.3 (4)	C32—C33—C34—C35	−1.5 (7)
C8—C9—C10—C4	−179.5 (3)	C33—C34—C35—C36	1.8 (6)
C8—C9—C10—C5	0.0 (4)	C34—C35—C36—C31	0.2 (6)
C9—C1—C2—C3	2.9 (4)	C36—C31—C32—C33	2.8 (6)
C9—C1—C2—C12	−177.7 (3)	C37—O2—C34—C33	0.8 (6)
C9—C8—C22—N1	179.9 (3)	C37—O2—C34—C35	−178.2 (3)
C10—C5—C6—O4	−177.2 (3)	C38—N2—C27—C18	170.9 (3)
C10—C5—C6—C7	1.5 (5)	C38—C39—C40—C41	−4.2 (7)
C10—C5—C23—C24	−127.8 (3)	C39—C38—C43—C42	−2.2 (6)
C10—C5—C23—C25	103.3 (4)	C39—C40—C41—O6	180.0 (4)
C11—C12—C13—C14	2.5 (5)	C39—C40—C41—C42	3.1 (6)
C11—C12—C13—C26	−179.6 (3)	C40—C41—C42—C43	−1.6 (6)
C11—C19—C20—C14	2.8 (4)	C41—C42—C43—C38	1.1 (6)
C11—C19—C20—C15	−175.6 (3)	C43—C38—C39—C40	3.7 (6)
C12—C2—C3—C4	179.7 (3)	C44—O6—C41—C40	175.3 (3)
C12—C2—C3—C21	2.9 (4)	C44—O6—C41—C42	−8.1 (5)

### Hydrogen-bond geometry (Å, º)

**Table:**

*D*—H···*A*	*D*—H	H···*A*	*D*···*A*	*D*—H···*A*
N1—H1*A*···O3	0.95 (5)	1.83 (5)	2.538 (4)	129 (4)
N2—H2···O7	0.92 (3)	1.87 (3)	2.550 (3)	129 (3)
O1—H1···O6^i^	0.91 (4)	2.13 (4)	2.912 (3)	144 (3)
O4—H4···O3	0.86 (3)	2.04 (4)	2.574 (4)	119 (3)
O5—H5···O3^ii^	0.82 (3)	1.98 (3)	2.684 (3)	143 (3)
O8—H8···O7	0.98 (3)	2.17 (3)	2.601 (3)	105 (2)
O8—H8···O7^iii^	0.98 (3)	1.83 (3)	2.757 (3)	158 (3)

Symmetry codes: (i) *x*−1, *y*, *z*; (ii) −*x*, −*y*+1, −*z*+1; (iii) −*x*+1, −*y*, −*z*.
